# Plasma Concentrations of High Mobility Group Box 1 Proteins and Soluble Receptors for Advanced Glycation End-Products Are Relevant Biomarkers of Cognitive Impairment in Alcohol Use Disorder: A Pilot Study

**DOI:** 10.3390/toxics12030190

**Published:** 2024-02-29

**Authors:** Fernando Rodríguez de Fonseca, Francisco Medina-Paz, Mira Sapozhnikov, Isaac Hurtado-Guerrero, Leticia Rubio, Stella Martín-de-las-Heras, Nerea Requena-Ocaña, María Flores-López, María del Mar Fernández-Arjona, Patricia Rivera, Antonia Serrano, Pedro Serrano, Sara C. Zapico, Juan Suárez

**Affiliations:** 1 Instituto de Investigación Biomédica de Málaga y Plataforma en Nanomedicina-IBIMA Plataforma BIONAND, 29590 Málaga, Spain; fernando.rodriguez@ibima.eu (F.R.d.F.); isaac.hurtado@uma.es (I.H.-G.); lorubio@uma.es (L.R.); smdelasheras@uma.es (S.M.-d.-l.-H.); nerera.requena@ibima.eu (N.R.-O.); maria.flores@ibima.eu (M.F.-L.); marfernandez@uma.es (M.d.M.F.-A.); patricia.rivera@ibima.eu (P.R.); antonia.serrano@ibima.eu (A.S.); pedro.serrano.c@gmail.com (P.S.); 2 Servicio de Neurología, Hospital Regional Universitario de Málaga, 29010 Málaga, Spain; 3 Department of Chemistry and Environmental Sciences, New Jersey Institute of Technology, Tiernan Hall 365, Newark, NJ 07102, USA; francisco.medinapaz@njit.edu (F.M.-P.); ms2948@njit.edu (M.S.); 4 Departamento de Anatomía Humana, Medicina Legal e Historia de la Ciencia, Facultad de Medicina, Universidad de Málaga, 29071 Málaga, Spain; 5 UGC Salud Mental, Hospital Regional Universitario de Málaga, 29010 Málaga, Spain; 6 Anthropology Department, National Museum of Natural History, Smithsonian Institution, 10th and Constitution Ave. NW, Washington, DC 20560, USA

**Keywords:** addiction, alcohol use disorder, Alzheimer’s disease, cognitive impairment, dementia, oxidative stress, HMGB1, advanced glycation end-products, apolipoprotein D

## Abstract

Alcohol use disorder (AUD) is a major component in the etiology of cognitive decline and dementia. Underlying mechanisms by which long-term alcohol abuse causes cognitive dysfunction include excessive oxidative stress and inflammation in the brain, activated by increased reactive oxygen/nitrogen species (ROS/RNS), advanced glycation end-products (AGEs) and high-mobility group box 1 protein (HMGB1). In a pilot study, we examine the potential clinical value of circulating biomarkers of oxidative stress including ROS/RNS, HMGB1, the soluble receptor for AGE (sRAGE), the brain biomarker of aging apolipoprotein D (ApoD), and the antioxidant regulator nuclear factor erythroid 2-related factor 2 (NRF2) as predictive indices for cognitive impairment (CI) in abstinent patients with AUD (*n* = 25) compared to patients with established Alzheimer’s disease (AD, *n* = 26) and control subjects (*n* = 25). Plasma concentrations of sRAGE were evaluated with immunoblotting; ROS/RNS with a fluorometric kit; and HMGB1, ApoD, and NRF2 by ELISA. Abstinent AUD patients had higher sRAGE, ROS/RNS (*p* < 0.05), and ApoD (*p* < 0.01) concentrations, similar to those of AD patients, and lower NRF2 (*p* < 0.01) concentrations, compared to controls. These changes were remarkable in AUD patients with CI. HMGB1, and sRAGE correlated positively with duration of alcohol use (rho = 0.398, *p* = 0.022; rho = 0.404, *p* = 0.018), whereas sRAGE correlated negatively with periods of alcohol abstinence (rho = −0.340, *p* = 0.045). A predictive model including ROS/RNS, HMGB1, sRAGE, alcohol use duration, and alcohol abstinence periods was able to differentiate AUD patients with CI (92.3% of correct predictions, ROC-AUC= 0.90) from those without CI. In conclusion, we propose ROS/RNS, HMGB1, and sRAGE as stress biomarkers capable of predicting cognitive impairment in AUD patients.

## 1. Introduction

Alcohol use disorder (AUD) is a chronic condition characterized by an excessive and uncontrollable consumption of alcohol, leading to physical and psychological dependence [1]. Long-term alcohol abuse can cause not only deterioration associated with thiamine deficiency, but also multiple medical comorbidities and a decline in cognitive abilities that interferes with a person’s daily functioning [2]. AUD is considered an established risk factor of early cognitive impairment and progression of certain types of dementia [3,4,5]. Alcohol-related dementia is primarily caused by the toxic, stressful, and inflammatory effects of alcohol on the brain, which typically affects memory, executive functioning, attention, and other cognitive abilities [4,6]. In addition, alcohol abuse can also worsen existing dementia or accelerate its progression in individuals diagnosed with Alzheimer’s disease (AD) [7,8].

The underlying mechanisms by which alcohol activate innate immune signaling and oxidative stress affecting cognitive functions are partially understood, despite the remarkable ability of alcohol to activate the Toll-like receptor (TLR) 4 directly [9,10,11,12]. Alcohol toxicity-induced oxidative stress and inflammation in the brain lead to neuronal cell death (intracellular damage and apoptosis) and impaired neuronal function (e.g., mitochondrial dysfunction), caused by the release of neuroimmune signaling, such as high-mobility group box 1 (HMGB1), as well as the accumulation of reactive oxygen and nitrogen species (ROS/RNS) and toxic protein aggregates, including advanced glycation end-products (AGEs) [13,14,15]. AGEs and HMGB1 that are passively accumulated from necrotic or damaged cells or actively secreted by cells under alcoholic conditions can activate the cell surface receptor for AGEs (RAGE) in neurons and glia [16].

RAGE is a member of the immunoglobulin superfamily and a type I transmembrane protein that binds to AGEs, HMGB1, lipopolysaccharides, S100, and amyloid beta, among others [17,18]. RAGE is highly expressed in neurons under pathological conditions, such as alcoholic brain damage, which is activated by macrophages (microglia), promoting the activation of the nuclear factor kappa-light-chain-enhancer of activated B cells (NF-κB p65) and stress-activated protein kinase (SAPK)/c-Jun N-terminal kinase (JNK)/p38 pathways [14,19,20]. Phosphorylated NF-κB p65 is increased by alcohol in brain and results in induction of pro-inflammatory gene transcription of cytokines, chemokines, and cell adhesion molecules and release of other signaling mediators of macrophage activation such as prostaglandins, nitric oxide, and oxygen free radicals [14,16,21]. Particularly, RAGE activation by HMGB1 was described to cause cell death through NF-κB pathways in alcohol brain damage and AD [22,23,24].

RAGE overactivity contributes to pathological changes in aging and age-related degenerative diseases, such as AD, Parkinson’s disease, and alcohol brain damage [14,25]. Preventing interactions between RAGE and its ligands (e.g., HMGB1) via a soluble decoy isoform of the receptor (soluble RAGE or sRAGE) has recently emerged as an endogenous antioxidant mechanism and a novel potential biomarker for early diagnosis and prognosis of a variety of RAGE-mediated disorders [14,15,17,26,27], including neurodegenerative disorders [28,29,30,31,32] and neuroinflammatory-related diseases [33,34,35]. Indeed, chronic alcohol consumption was described to increase sRAGE levels through matrix metalloproteolytic (MMP)-9 activity [36].

Since AUD is the most important risk factor for early dementia through progressive neuronal injury, excessive oxidative stress, and neuroinflammation across the lifespan [5,37], there is a need to identify reliable biomarkers that can help detect and diagnose alcohol-induced brain damage early, before the onset of complicating clinical symptoms. Here, we investigated the contributing role of the HMGB1–sRAGE axis in alcohol-induced cognitive dysfunction. To this aim, we evaluated whether plasma concentrations of sRAGE, HMGB1, and ROS/RNS, together with the brain biomarker of aging apolipoprotein D (ApoD) and the antioxidant regulator nuclear factor erythroid 2-related factor 2 (NRF2), are predictive indices for cognitive impairment in abstinent AUD patients compared to AD patients and control subjects.

## 2. Materials and Methods

### 2.1. Ethics Statement

Informed consent was obtained from all subjects after receiving a complete description of the study. The study approved by the Clinical and Ethics Research Committee of Hospital Regional Universitario de Malaga-PEIBA (PNSD 2019/040, code number: 0681-N-21, date: 14 April 2021) and the NJIT (IRB protocol number: 2110013076), in accordance the Ethical Principles for Medical Research with Human Subjects adopted in the Declaration of Helsinki (64th General Assembly of the World Medical Association, Fortaleza, Brazil, October 2013) and the Spanish law on data protection (Regulation 2016/679 of the European Parliament and the Council of 27 April 2016 on the protection of natural persons with regard to the processing of personal data and the free circulation of such data), and repealing Directive 95/46/EC (General Data Protection Regulation). All data collected were given code numbers to maintain privacy and confidentiality.

### 2.2. Recruitment and Screening of Participants

The present cross-sectional study included 25 patients with AUD in outpatient treatment, in abstinence for at least 2 weeks (accredited by self-report and alcohol monitorization in breath); 26 patients with AD from neurology outpatient settings; and 25 healthy control subjects without the presence of cognitive impairment, medical illnesses, or substance use disorders. AUD patients were collected at the Provincial Center of Drug Dependence (Málaga, Spain). AD patients were recruited from the Neurology Service of the Hospital Regional Universitario de Málaga (Málaga, Spain). Control participants were obtained from the databases of healthy subjects of the National DNA Biobank (Valencia, Spain).

The AUD group met the following inclusion criteria: persons over 18 years of age in the abstinence period (>2 weeks) with a diagnosis of AUD, who were in outpatient treatment and willing to participate by signing the informed consent form. Exclusion criteria were abnormal liver function blood tests (aspartate aminotransferase (AST), alanine transaminase (ALT), gamma-glutamyl transferase (GGT), or alkaline phosphatase (ALP)), treatment with anti-inflammatory drugs, clinical history of long-term inflammatory disease or cancer, severe language limitations, pregnant or breast-feeding women, and infectious diseases such as Hepatitis C Virus (HCV), Hepatitis B Virus (HBV), and Human Immunodeficiency Virus (HIV). The AD group was included as a clinical control of the AUD group because of known cognitive impairment. The AD group met the following inclusion criteria: persons older than 60 years of age under neurologic treatment with a Montreal of Cognitive Assessment (MoCA) score of less than 25 and willingness to participate by signing the informed consent form. Exclusion criteria included clinical history of alcohol consumption during the last year with a score above 8 on the AUD Identification Test (AUDIT), severe language limitations, and infectious diseases such as HCV, HBV, and HIV. As for the control group, participants with a history of substance abuse, comorbid psychiatric disorders, medical illness, and cognitive impairment were not included. Following inclusion and exclusion criteria, individuals for controls (52.48 ± 2.26 years of age) and AD (77.07 ± 4.65 years of age) were also selected with respect to the mean age of the AUD group (42.07 ± 12.45 years of age) and to obtain representation of the female population in each group.

### 2.3. Psychiatric and Neuropsychological Evaluation

The Spanish version of the Psychiatric Research Interview for Substance and Mental Diseases (PRISM diagnostic interview) was used for the assessment of AUD and other psychiatric disorders according to DSM-5-TR (Diagnostic and Statistical Manual of Mental Disorders, 5th edition) criteria. The PRISM is a semi-structured interview with good psychometric properties in the assessment of AUD and major comorbid psychiatric disorders related to substance abuse [38,39].

Neuropsychological evaluation was performed using the Montreal of Cognitive Assessment (MoCA) for diagnosis related to general cognitive status, which has demonstrated reliability and good psychometric properties. We defined a score value of 23–26 as the cut-off point for mild cognitive impairment and a score value of <22 as the cut-off point for moderate-severe cognitive impairment, as reported in previous studies [40].

All psychiatric and neuropsychological evaluations were performed in the morning. The clinicians strictly followed a specified assessment design so that the tests would not interfere with each other and to reduce the inter-clinician variability in testing. The tests were performed in order: (1) sociodemographic and drug screening (PRISM) and (2) MoCA. The duration of the session was estimated at 90 min.

### 2.4. Sample Collection

Blood samples were obtained in the morning (08:00–10:00 a.m.) after an 8–12 h fasting period, and before psychiatric interviews. Venous blood was drawn into 10 mL K2 EDTA tubes (BD, Franklin Lakes, NJ, USA) and processed immediately to obtain plasma. Blood samples were centrifuged at 2200× *g* for 15 min (4 °C). Finally, plasma samples were recorded and stored at −80 °C when not in use.

### 2.5. Protein Quantification and Immunoblotting for sRAGE

Plasma samples were diluted in phosphate-buffered saline (PBS) to obtain a final dilution of 1:100. Plasma proteins were quantified using a Qubit^®^ Quant-iT™ Protein assay kit and a Qubit^®^ fluorometer (Invitrogen Life Technologies, Waltham, MA, USA), according to the manufacturer’s protocol.

Samples containing equivalent amounts of total protein (50 µg) were diluted 1:4 in Laemmli 4× loading buffer (Bio-Rad Laboratories Inc., Hercules, CA, USA). Samples were loaded onto 4–20% precast polyacrylamide Mini-PROTEAN^®^ TGX™ gels with 1× Tris/Glycine running buffer (Bio-Rad Laboratories Inc., Hercules, CA, USA). Plasma proteins were run at 100 V for about 1 h.

The proteins in gels were then transferred to Bio-Rad Immun-BLOT^®^ PVDF pre-cut membranes using a Bio-Rad Trans-Blot^®^ Turbo™ transfer system. Each membrane was stained with Ponceau S stain (Thermo Fisher Scientific Inc., Waltham, MA, USA) and then washed in Tris-buffered saline with Tween^®^ 20 (TBS-T) (Sigma-Aldrich, Burlington, MA, USA). The membranes were pre-incubated with EveryBlot blocking buffer (Bio-Rad) for 5 min, according to the manufacturer’s protocol. The respective blotted membrane lines were then incubated separately with rabbit monoclonal antibodies against RAGE and transferrin (cat. no. A23422 and A1448, respectively, ABclonal Technology, Woburn, MA, USA), diluted at 1:1000 in Bio-Rad EveryBlot blocking buffer, overnight at 4 °C. After washing for 25 min in TBS-T, the membranes were incubated with anti-rabbit secondary antibody (cat. no. AS029, ABclonal Technology), diluted at 1:2000 in Bio-Rad EveryBlot blocking buffer, at room temperature for one hour, and washed for 25 min in TBS-T.

Specific protein bands were stained using Prometheus™ ProSignal^®^ Pico ECL reagent (Genesee Scientific Co., El Cajon, CA, USA) and visualized with a C-DiGit^®^ Blot Scanner (LI-COR Biosciences, Inc., Lincoln, NE, USA). Western blots showed that each primary antibody detected a protein of the expected molecular size. Optical density quantification of each band was performed using ImageJ software (NIH). Results are expressed as RAGE/transferrin ratio.

### 2.6. Oxidative Stress Analysis

OxiSelect™ In Vitro ROS/RNS assay kit (Green Fluorescence) based on 2,7-dichlorodihydrofluorescein diacetate (DCFH) was used to analyze total reactive oxygen species and reactive nitrogen species, including hydrogen peroxide, nitric oxide, peroxyl radical, and peroxynitrite anion, following manufacturer’s instructions (cat. no. STA-347; Cell Biolabs, Inc., San Diego, CA, USA). Plasma samples (50 µL) were diluted at 1:10. Fluorescence was measured in a Tecan microplate reader (Tecan Group Ltd., Männedorf, Zürich, Switzerland) at 480 nm for excitation and 530 nm for emission. Concentrations (nM) were plotted relative to control.

### 2.7. Human High-Mobility Group Box 1 Protein ELISA

Plasma concentration of HMGB1 was measured using human HMGB1 ELISA kit (cat. no. NBP2-62766, Novus Biologicals, Centennial, CO, USA) following the manufacturer’s instructions. Plasma samples (30 µL), diluted at 1:3, were measured by colorimetry in a GloMax^®^ Discover microplate reader (Promega Biotech Ibérica S.L., Alcobendas, Spain) at the wavelength of 450 nm. Concentrations were expressed in pg/mL.

### 2.8. Apolipoprotein D ELISA

Plasma concentration of human ApoD was measured using the human apolipoprotein D ELISA kit (cat. no. A78996, Antibodies.com LLC, St. Louis, MO, USA) following the manufacturer’s instructions. Plasma samples (100 µL), diluted at 1:10, were measured by colorimetry in a Tecan^®^ microplate reader (Tecan Group Ltd., Männedorf, Zürich, Switzerland) capable of measuring absorbance at 450 nm. Concentrations were expressed in ng/µL.

### 2.9. Human Nuclear Factor Erythroid 2-Related Factor 2 ELISA

Plasma concentration of human NFR2 was measured using the human NFE2L2 ELISA kit (ref. no. CSB-EL015752HU; Cusabio, Houston, TX, USA) following the manufacturer’s instructions. Plasma samples (100 µL), diluted at 1:10, were measured by colorimetry in a GloMax^®^ Discover microplate reader (Promega Biotech Ibérica S.L.) at the wavelength of 450 nm. Concentrations were expressed in pg/mL.

### 2.10. Statistical Analysis

Data in tables and graphs are expressed as numbers and percentage of subjects [N (%)] or means and standard deviations (mean ± SD). A Lilliefors-corrected Kolmogorov–Smirnov test was used to assess the normal distribution of the variables. Significance of differences in qualitative and quantitative variables was determined by Fisher’s exact test (Chi-square), Kruskal–Wallis test, or ANOVA test, when appropriate. Multiple analysis of covariance (ANCOVA) was used to analyze the relative effects of explanatory variables (i.e., cognitive impairment) on plasma concentrations of HMGB1, RAGE, ROS/RNS, ApoD, and NRF2 controlling for age (when it was appropriated). Multiple comparisons were performed using the Games–Howell post-hoc test, when appropriate. Estimation of effect size was measured using partial eta squared (η_p_^2^). When main effects of ANCOVA were not detected, single effects by Student’s *t* test or Mann–Whitney test were analyzed, when appropriate. Spearman’s coefficient (rho) was used to perform correlation analyses. Binary logistic regression analysis was performed using Pearson’s Chi-square (χ^2^) test with the Hosmer–Lemeshow test. The receiver operating characteristic (ROC) curve was used to determine the threshold for discrimination of cognitive impairment in AUD. Statistical analyses were carried out using GraphPad Prism version 9, JASP version 0.18, and IBM SPSS Statistic version 28 (IBM, Armonk, NY, USA). A *p*-value of less than 0.05 was considered statistically significant.

## 3. Results

### 3.1. Sociodemographic and Clinical Characteristics of AUD, AD, and Control Groups

The three groups matched for body mass index and sex, but significant difference was found for age (*p* < 0.001) due to the older age of the AD group (Table 1).

Among the clinical characteristics of patients attending outpatient treatment for AUD (Table 2), exclusive alcohol consumption was the most prevalent (52%), followed by combination of alcohol with cocaine and cannabis (20%), alcohol with cocaine (16%), and alcohol with cannabis (12%). The variables defining the AUD group indicated that the mean age of first alcoholic drink (alcohol use onset) was 15 years, whereas the mean age of alcohol dependence onset was 22 years, with 15 years of problem drinking (duration of use) and a duration of 476 days of abstinence (range: 1 to 4380 days) at the time of evaluation. Prevalence of comorbid psychiatric disorders was common in AUD patients, with mood and anxiety disorders being the most commonly diagnosed (56% and 48%, respectively), followed by personality and psychotic disorders (28% and 8%, respectively). Neuropsychological assessment indicated that 68% of patients with AUD had some degree of cognitive impairment (CI) assessed by MoCA.

Among the clinical characteristics of AD patients (Table 3), dementia related to degenerative and amnesic etiology was the most prevalent (34.6%), followed by amnesic etiology (23%) and degenerative etiology (15.4%). Most AD patients had a high neurodegenerative probability (38.5%), while 30.8% had a low neurodegenerative probability, and 19.2% had a medium neurodegenerative probability. Neurodegenerative assessment indicated that AD patients had some level of dementia, assessed by the Blessed dementia test, with 80.8% having low level of dementia, 73% having some episodic memory impairment, and 73% having some CI.

Significant differences in CI were found when comparing AD and AUD groups (Table 4). Controlling for age, MoCA scores were lower in AD patients than AUD patients (*p* < 0.001), suggesting higher CI in AD patients.

**Figure 1:**
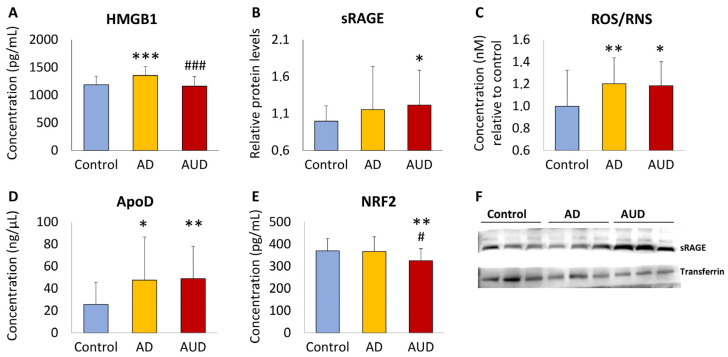
Plasma concentration of high-mobility group box 1 protein ((**A**): HMGB1), soluble receptor for advanced glycation end-products ((**B**): sRAGE), reactive oxygen/nitrogen species ((**C**): ROS/RNS), apolipoprotein D ((**D**): ApoD), and nuclear factor erythroid 2-related factor 2 ((**E**): NRF2), according to the control, Alzheimer’s disease (AD), and alcohol use disorder (AUD) groups. Representative western blots of the sRAGE protein have been included in panel (**F**). Data were analyzed by Games–Howell post-hoc test or Student’s *t* test (single effect in (**B**)), when appropriate. Bars represent means ± standard deviations. Symbols: *^/^**^/^*** *p* < 0.05/0.01/0.001 versus control group; ^#/###^ *p* < 0.05/0.001 versus AD group.

We analyzed the plasma concentrations of HMGB1, sRAGE, ROS/RNS, ApoD, and NRF2 in the AUD patients, compared to those of AD patients and control subjects (Figure 1). Using one-way ANCOVA with group as a factor and age as a controlling covariate, we observed that plasma concentrations of HMGB1, sRAGE, ROS/RNS, ApoD, and NRF2 were different between groups (F_2,75_ = 4.786, *p* = 0.011, η_p_^2^ = 0.113; F_2,77_ = 2.508, *p* = 0.08, η_p_^2^ = 0.061; F_2,77_ = 6.482, *p* = 0.003, η_p_^2^ = 0.144; F_2,77_ = 6.124, *p* = 0.003, η_p_^2^ = 0.137; F_2,77_ = 3.902, *p* = 0.024, η_p_^2^ = 0.092, respectively). Plasma HMGB1 and NRF2 concentrations were lower in the AUD group compared to those of the AD group (^#/###^ *p* < 0.05/0.001; Figure 1A,E). However, plasma sRAGE, ROS/RNS, and ApoD concentrations did not differ significantly between the AUD and AD groups (Figure 1B–D). The AUD group had higher sRAGE, ROS/RNS, and ApoD concentrations and lower NRF2 concentrations, compared to the control group (*^/^** *p* < 0.05/0.01; Figure 1B–E). In addition, the AD group had higher HMGB1, ROS/RNS, and ApoD compared to the control group (*^/^**^/^*** *p* < 0.05/0.01/0.001; Figure 1A,C,D).

### 3.2. Cognitive Impairment in AUD Patients Alters Plasma Concentrations of HMGB1, RAGE, ROS/RNS, ApoD, and NRF2 Compared to AD and Control Groups

**Figure 2:**
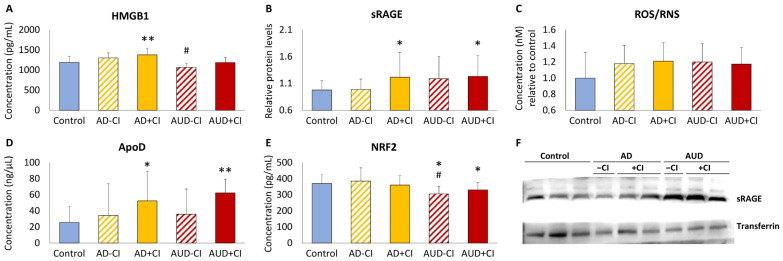
Plasma concentration of high-mobility group box 1 protein ((**A**): HMGB1), soluble receptor for advanced glycation end-products ((**B**): sRAGE), reactive oxygen/nitrogen species ((**C**): ROS/RNS), apolipoprotein D ((**D**): ApoD), and nuclear factor erythroid 2-related factor 2 ((**E**): NRF2), according to the control, Alzheimer’s disease (AD), and alcohol use disorder (AUD) subgroups with (+) and without (−) cognitive impairment (CI). Representative western blots of the sRAGE protein have been included in panel (**F**). Data were analyzed by Games–Howell post-hoc test or Student’s *t* test (single effect in (**B**,**E**), when appropriate. Bars represent means ± standard deviations. Symbols: *^/^** *p* < 0.05/0.01 versus control group; ^#^ *p* < 0.05 versus respective AD-CI and AD+CI subgroups.

We analyzed whether presence of CI assessed by MoCA alters plasma levels of HMGB1, sRAGE, ROS/RNS, ApoD, and NRF2 in the AUD group, compared to those of the AD and control groups (Figure 2). Using one-way ANCOVA with CI (presence and absence) in each group as a factor and age as a controlling covariate, we observed that plasma concentrations of HMGB1, sRAGE, ROS/RNS, ApoD, and NRF2 were different between subgroups (F_4,73_ = 4.098, *p* = 0.005, η_p_^2^ = 0.183; F_4,73_ = 1.944, *p* = 0.112, η_p_^2^ = 0.094; F_4,75_ = 3.850, *p* = 0.007, η_p_^2^ = 0.170; F_4,75_ = 4.068, *p* = 0.005, η_p_^2^ = 0.178; F_4,75_ = 2.275, *p* = 0.069, η_p_^2^ = 0.108, respectively). AUD patients without CI (AUD-CI subgroup) had lower plasma concentrations of HMGB1 and NRF2 compared to the AD-CI subgroup (^#^ *p* < 0.05; Figure 2A,E), as well as lower plasma NRF2 concentrations compared to control group (* *p* < 0.05; Figure 2E). CI in AUD and AD patients (AUD+CI and AD+CI subgroups) was associated with higher plasma concentrations of HMGB1, sRAGE, and ApoD, compared to the control group (*^/^** *p* < 0.05/0.01; Figure 2A,B,D).

### 3.3. Comorbid Psychiatric Disorders Alter the Effect of Cognitive Impairment on Plasma Concentrations of sRAGE in AUD Patients

We investigated whether comorbid mood and anxiety disorders influence the effect of CI on plasma concentrations of HMGB1, RAGE, ROS/RNS, ApoD, and NRF2 in AUD patients. Using one-way ANCOVA with CI and comorbid mood disorder or comorbid anxiety disorder as factors and age as a covariate, we found an interaction between CI and comorbid anxiety disorder (F_1,25_ = 4.103, *p* = 0.05, η_p_^2^ = 0.141) and an interaction between CI and comorbid mood disorder (F_1,25_ = 6.432, *p* = 0.018, η_p_^2^ = 0.205) in the plasma concentrations of sRAGE in AUD patients. Thus, the effect of CI on sRAGE depends on the presence of comorbid anxiety and mood disorders in AUD patients. In addition, the effect of CI on the plasma HMGB1 concentrations remained significant when controlling for comorbid mood disorder (F_1,23_ = 4.316, *p* = 0.049, η_p_^2^ = 0.152), and near significant when controlling for comorbid anxiety disorder (F_1,24_ = 3.591, *p* = 0.07, η_p_^2^ = 0.130) in AUD patients.

### 3.4. Correlation Analysis of HMGB1, sRAGE, ROS/RNS, ApoD, and NRF2 According to Variables of AUD Severity

Partial correlations controlling for age were analyzed to explore whether changes in plasma concentrations of HMGB1, sRAGE, ROS/RNS, ApoD, and NRF2 are significantly related to variables of AUD severity including age of alcohol use onset, age of alcohol dependence onset, duration of alcohol use, duration of alcohol abstinence, and periods of alcohol abstinence. Interestingly, plasma HMGB1 and sRAGE concentrations correlated positively with duration of alcohol use (rho = 0.398, *p* = 0.022; rho = 0.404, *p* = 0.018, respectively). We also found positive correlations between HMGB1 concentration and cocaine use duration (rho = 0.339, *p* = 0.039), and between HMGB1 and NRF2 concentrations (rho = 0.381, *p* = 0.023). Interestingly, sRAGE correlated negatively with periods of alcohol abstinence (rho = −0.340, *p* = 0.045). We also observed a negative correlation between ROS/RNS and age of alcohol use onset (rho = −0.360, *p* = 0.039) and positive correlations between ApoD and alcohol abstinence duration (rho = 0.411, *p* = 0.023) and between ApoD and cocaine dependence onset (rho = 0.503, *p* = 0.048). See Supplementary Table S1 for complete correlation analysis.

### 3.5. Predictive Variables of Cognitive Impairment in AUD Patients

**Figure 3:**
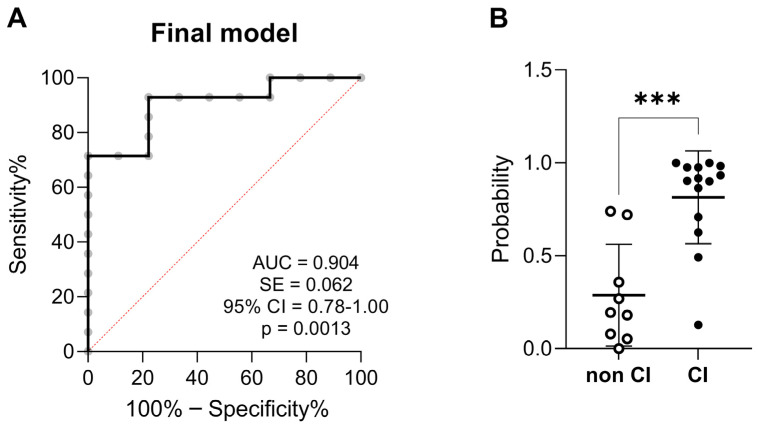
ROC analysis and scatter dots for multivariate predictive of final model of cognitive impairment in the AUD group. (**A**) ROC curve for the final model whose variables were HMGB1, sRAGE, ROS/RNS, age of alcohol dependence onset, and periods of alcohol abstinence. (**B**) Scatter plot of the predictive probabilities of the final model. Mean values ± SD are represented in the scatter plot. AUD patients without cognitive impairment (CI) are represented in open circles. AUD patients with CI are represented in filled black circles. AUC, area under the curve; SE, standard error; 95% CI, 95% confidence interval. Symbol: *** *p* < 0.001.

A binary logistic regression model was performed to discriminate between AUD patients with and without CI. The first-step variables introduced in the model were HMGB1, sRAGE, ROS/RNS, ApoD, NRF2, age of alcohol use onset, age of alcohol dependence onset, duration of alcohol use, duration of alcohol abstinence, and periods of alcohol abstinence. Using a backward stepwise method (Wald test) of the model, the predicted covariables were restricted to HMGB1, sRAGE, ROS/RNS, age of alcohol dependence onset, and periods of alcohol abstinence. The Hosmer–Lemeshow test indicated good calibration (χ^2^ = 0.882, *p* = 0.348). The final model was able to explain the variation of CI in 63.8% of the subjects according to the Nagelkerke R^2^ method. The final model had a sensitivity (true positive rate) and a specificity (false negative rate = 100% − specificity%) to correctly predict AUD patients with and without CI in a 92.3% and 77.8%, respectively, of cases (overall % correct = 86.36%, cut-off value = 0.5). ROC analysis (AUC = 0.904, 95% confidence interval = 0.79–1.00) indicated a high discrimination power of those AUD patients with CI (Figure 3A). The predicted probability also indicated significant differences between AUD patients with and without CI (U = 12, *p* < 0.001; Figure 3B).

## 4. Discussion

The World Health Organization considers alcohol consumption a main component in the etiology of dementia [41]. Alcohol-related dementia was described as the most prevalent diagnosis of early (under 65 years of age) onset dementia [42] and heavy alcohol consumption was associated with greater cognitive impairment in older adults [43]. Early detection and monitoring of molecular mechanisms leading to alcohol impairment of cognitive functions remain a major challenge for predictive diagnosis and prognosis in clinical practice. In this regards, oxidative stress and inflammation are key underlying mechanisms in the brain that can be explored to identify stress biomarkers capable of predicting cognitive dysfunctions in patients with AUD [9,10,11,12].

The present study examined plasma concentrations of sRAGE, HMGB1, and ROS/RNS, together with the brain biomarker of aging ApoD and the antioxidant regulator NRF2 in AUD patients, who were recruited from active outpatient treatment programs. The abstinent AUD patients were clinically characterized through psychiatric interviews based on the DSM-5-TR and main AUD severity-related variables (e.g., alcohol consumption onset, alcohol dependence onset, alcohol abstinence duration and periods, and psychiatric disorders, among others). The main findings indicate that abstinent AUD patients had higher plasma sRAGE, ROS/RNS, and ApoD concentrations, similar to those of AD patients, and lower NRF2 concentrations compared to controls. These changes were more significant in AUD patients with CI. We also evaluated the relationship between these plasma biomarkers and AUD severity-related variables. We found that HMGB1 and sRAGE were positively correlated with duration of alcohol use, whereas sRAGE was negatively correlated with periods of alcohol abstinence. These results suggest that sRAGE, by acting as a decoy receptor that protects against exacerbated inflammatory response to HMGB1 [17,26], is highly associated with alcohol dependence (increased) and abstinence (decreased). Interestingly, elevated ROS/RNS levels were negatively correlated with the onset of alcohol consumption, suggesting that AUD patients starting alcohol consumption at early ages may have higher oxidative damage that contributes to neuronal cell death [44]. In addition, ApoD was positively correlated with duration of alcohol abstinence, suggesting a glial cell-associated regenerative function and reduced sensitivity to oxidative stress following alcohol-induced brain damage [45,46]. To identify reliable biomarkers of alcohol-induced neurocognitive disorder, a binary logistic regression analysis was performed to predict CI in AUD patients. A model including ROS/RNS, HMGB1, sRAGE, alcohol use duration, and alcohol abstinence periods was able to stratify the AUD sample in cognitively impaired and non-cognitively impaired patients by 92.3% of correct predictions. Finally, we also found that comorbid mood and anxiety disorders in AUD patients influence the effect of CI on plasma concentrations of sRAGE. These results suggest that sRAGE plays a role in comorbid psychiatric disorders, contributing to cognitive dysfunction in patients with AUD.

A noteworthy aspect of the present study is the not-well-known effects of alcohol use on plasma sRAGE levels, which were partially present in the present study. sRAGE was proposed to cause loss of cell surface RAGE/NF-κB-induced pro-inflammatory signaling by preventing binding to ligands, such as HMGB1, AGEs, S100B, β-amyloid, and LPS, among others [47,48,49,50]. Low levels of sRAGE and high levels of HMGB1 were associated with further pro-inflammatory and pro-oxidant signaling [32]. In fact, HMGB-1 and sRAGE have been described to correlate inversely in the blood circulation of healthy individuals [51]. As a brain aging hallmark under pathological conditions, reduced levels of sRAGE have been specifically described to be a risk biomarker of neurodegenerative conditions [52], including mild cognitive impairment, early cognitive decline in type-2 diabetes, AD, and vascular dementia, among others [28,29,30,31,32,53]. However, increased sRAGE levels were also described to prevent harmful effects of HMGB1–RAGE interaction as an antioxidant response to the amplification of pathological pro-inflammatory processes and exacerbated oxidative stress conditions [31,54]. Interestingly, a recent study demonstrated that chronic alcohol consumption was described to reduce RAGE protein expression in the alveolar macrophage and to increase sRAGE levels in the alveolar lavage, probably through increased MMP-9 activity [36]. This agrees with our results, which indicated elevated sRAGE levels in AUD patients and in AUD patients with cognitive impairment, similar to those of AD patients with cognitive impairment. However, severity of cognitive impairment did not differentiate sRAGE levels in the AUD subgroups. Our results also indicated that plasma sRAGE levels positively correlated with the duration of alcohol use, as well as negatively with the periods of alcohol abstinence in the AUD patients. Although future studies should investigate in detail the antioxidant role of sRAGE in preventing HMGB1–RAGE-induced pro-inflammatory processes and exacerbated oxidative stress, the findings suggest that sRAGE was significantly affected by alcohol use and abstinence and should be considered as a potential biomarker of ethanol toxicity-induced cognitive impairment.

The effects of alcohol on cognitive impairment and psychiatric comorbidities, including mood and anxiety disorders, were described to be mediated by novel biomarkers involving deficits in trophic signaling (BDNF, IGF1, and TGF-β1), disruption in the blood–brain barrier (VEGFA), and increased chemotaxis response (SDF-1, eotaxin, MIP-1α, MCP-1, and fractalkine) and brain damage (neurofilament light chain protein) that may contribute to neuroinflammation, oxidative stress, and cell death [40,55,56,57,58]. Indeed, a recent study proposes that vascular endothelial growth factor A (VEGFA) may mediate the effects of alcohol on cognitive impairment through blood–brain barrier permeability, facilitating the chemotactic infiltration (MIP-1α) of immune cells into the brain [55]. In addition, bioactive lipids such as lysophosphatidic acid species, which are involved in neurodevelopment and neural plasticity, have also been described to be vulnerable factors to excessive alcohol consumption involved in cognitive impairment and emotional behavior [58]. In the present study, we suggest that the role of sRAGE in cognitive impairment may be influenced by the presence of comorbid psychiatric disorders (e.g., mood and anxiety) in AUD patients, although larger cohorts are needed to confirm this relevant observation.

Alcohol-induced neuroimmune signaling is a complex mechanism by which multiple cytokines, chemokines, and other inflammatory molecules, such as HMGB1, converge on NF-κB intracellular cascades to activate, amplify, and expand oxidative stress and neuroinflammation [14,23]. The direct release of HMGB1 from excited neurons by adjacent glial induction of innate immune molecules stimulates RAGE or TLR4 and exacerbates neuronal network disruption and excitotoxic neuronal death [16,59,60]. Previous studies reported high immunohistochemical levels of HMGB1 in the cortex and cerebellum of mice after ethanol exposure and in postmortem in the human alcoholic brain [61,62]. Recent studies also support the notion that alcohol-induced activation of brain neuroimmune signaling is produced by the peripheral release of HMBG1 (e.g., from gut) and circulating pro-inflammatory cytokines (IL-6 and IL-17A) associated with alcohol-induced liver and pancreatic diseases [63] that may be actively transported across the blood–brain barrier. In agreement, our results indicated that plasma HMGB1 concentrations positively correlated with the duration of alcohol use in AUD patients. However, no alteration of HMGB1 was found in AUD patients during abstinence or associated with cognitive impairment, a finding that contrasts with the elevated HMGB1 concentrations in AD patients and AD patients with cognitive impairment. Thus, although HMGB1 has been identified as a critical immune mediator in alcohol abuse, there is a need to further investigate other mechanisms underlying RAGE activation in abstinent AUD patients with cognitive impairment. Therefore, future studies should focus on inflammatory molecules other than HMGB1, such as toxic protein aggregates, including AGEs.

Microglia activation via TLRs also produces oxidative stress in neurons by increasing AGEs, ROS, nitric oxide, and oxygen free radicals, while decreasing levels of the antioxidant regulator NRF2, the antioxidant glutathione, and antioxidative enzymes, such as glutathione peroxidase, among others [23]. In addition, ROS is a natural by-product of alcohol metabolism that increases mitochondria respiration and creates an exacerbated oxidative stress environment in the brain that contributes to neuronal cell death [44]. Our results indicated elevated ROS/RNS concentrations in the plasma of AUD patients during abstinence, the levels of which were similar to those in AD patients. In addition, it is noteworthy that plasma ROS/RNS levels are negatively correlated with age of alcohol use onset in AUD patients, suggesting that younger alcohol users may have exacerbated oxidative stress in the brain even through abstinence periods. However, these high levels of ROS/RNS appear not to be associated with cognitive impairment in the AUD and AD groups. These results should be corroborated by increasing the sample size and assessing specific neurocognitive functions, such as verbal episodic memory, executive function, visual memory, short-term memory, and verbal working memory, as was done in previous studies [40].

Alcohol was previously described to be positively related to apolipoprotein D in men, but not women [64]. Additional studies later reported that ApoD was down-regulated or up-regulated in the prefrontal cortex of different alcoholic groups, displaying some deficits in executive function and memory, as well as up-regulated in the motor cortex of chronic alcoholic subjects [65]. Although the involvement of ApoD as a biomarker of brain aging has been described in *APOE ε4*-related oxidative damage, cognitive impairment, and AD risk [66], to our knowledge, alcohol effects on ApoD have not been studied in abstinent AUD patients with cognitive impairment. Our results indicated higher plasma ApoD concentrations in AUD patients, similar to those of AD patients, and in AUD patients with cognitive impairment, similar to those of AD patients with cognitive impairment. Moreover, plasma ApoD concentrations were positively correlated with the duration of alcohol abstinence, suggesting a function of neuronal preservation and protection in response to decreasing oxidative stress and inflammation during alcohol withdrawal [45,46]. Nevertheless, further research is necessary to better understand the underlying mechanism of oxidative stress activated by alcohol that associate ApoD with cognitive impairment.

## 5. Conclusions and Limitations

The present data support the idea that circulating sRAGE appears to be highly associated with alcohol dependence and abstinence, probably through its role in controlling excessive neuroinflammation and oxidative damage induced by heavy and/or prolonged alcohol consumption that ultimately leads to deterioration of cognitive function. In the present study, we propose that ROS/RNS, HMGB1, and sRAGE may serve as reliable plasma biomarkers for predicting alcohol-induced cognitive impairment, especially in patients with AUD.

Limitations of the present study that should be addressed in future research include (1) a larger size sample to stratify the AUD group based on sex differences and comorbid use of other drugs (cocaine, cannabis, medication); (2) a longitudinal analysis of the effects of acute alcohol use versus chronic alcohol use on plasma sRAGE and HMGB1 concentrations; (3) further exploration of oxidative stress and inflammatory signaling as relevant biomarkers of psychiatric comorbidities in AUD; and (4) an integrative analysis of alcohol’s effects on additional RAGE ligands, including AGE species (e.g., the n(6)-carboxymethyllysine glycated protein), S100B, amyloid-β-proteins, Mac-1, and phosphatidylserine, among others, as well as putative AGE receptors, including macrophage scavenger receptors (SR-A), oligosaccharyl transferase-4 (AGE-R1), proteinkinase C substrate (AGE-R2), and galectin-3 (AGE-R3), that may play a role in AGE signaling or clearance associated with various chronic diseases.

## Figures and Tables

**Table 1:** Socio-demographic characteristics of the sample.

Total Sample (N = 76)					
Variables		Control Group (N = 25)	AD Group (N = 26)	AUD Group (N = 25)	*p*-Value
Age (mean ± SD)	Years	52.48 ± 2.26	77.07 ± 4.65	42.07 ± 12.45	<0.001 ^a^
Body mass index (mean ± SD)	Kg/m^2^	27.89 ± 3.90	27.69 ± 2.53	25.59 ± 5.87	0.107 ^b^
Sex [N (%)]	Women Men	8 17	14 12	7 18	0.053 ^c^

^a^ Kruskal–Wallis test. ^b^ ANOVA. ^c^ Chi-squared test. Bold values are statistically significant.

**Table 2:** Clinical characteristics of the AUD group.

Variables		AUD Group
Substance use [N (%)]	Alcohol	13 (52)
	Alcohol + cocaine	4 (16)
	Alcohol + cannabis	3 (12)
	Alcohol + cocaine + cannabis	5 (20)
AUD severity (mean ± SD)	Age (years) of use onset	14.85 ± 4.21
	Age (years) of dependence onset	21.96 ± 6.99
	Duration (years) of use	15.18 ± 13.16
	Duration (days) of abstinence	475.72 ± 1152
	Periods of abstinence	1.85 ± 2.10
Comorbid psychiatric disorders [N (%)]	Mood	14 (56)
	Anxiety	12 (48)
	Personality	7 (28)
	Psychotic	2 (8)
Cognitive impairment (MoCA ^a^) [N (%)]	Yes	17 (68)
	No	8 (32)

^a^ Montreal of Cognitive Assessment.

**Table 3:** Clinical characteristics of the AD group.

Variables		AUD Group
Dementia etiology * [N (%)]	Degenerative	4 (15.38)
	Amnesic	6 (23.08)
	Degenerative + amnesic	9 (34.61)
	Unknown	4 (15.38)
Neurodegenerative probability * [N (%)]	High	10 (38.46)
	Medium	5 (19.23)
	Low	8 (30.77)
Blessed dementia test [N (%)]	Severe-moderate	5 (19.23)
	Mild	21 (80.77)
Episodic memory test [N (%)]	Affected	2 (7.69)
	Mild	17 (65.38)
	No	7 (26.92)
Cognitive impairment (MoCA) * [N (%)]	Yes	19 (73.08)
	No	7 (26.92)

* Several data were missed in the clinical evaluation.

**Table 4:** Difference of cognitive impairment between AD and AUD.

Cognitive Impairment	AD	AUD	*p*-Value
MoCA score (mean ± SD)	23.04 ± 3.24	26.00 ± 3.82	<0.001 ^a^

^a^ Student’s *t* test (Shapiro–Wilk normality test, *p* = 0.1).

## Data Availability

The data presented in this study are available on request from the corresponding authors.

## Supplementary Materials

The following supporting information can be downloaded at: https://www.mdpi.com/article/10.3390/toxics12030190/s1, Table S1: Spearman’s partial correlations controlling for age between plasma concentrations of HMGB1, sRAGE, ROS/RNS, ApoD, and NRF2 and variables of AUD severity.
