# Supplementary material for: Plasma Concentrations of High Mobility Group Box 1 Proteins and Soluble Receptors for Advanced Glycation End-Products Are Relevant Biomarkers of Cognitive Impairment in Alcohol Use Disorder: A Pilot Study
Source: Toxics. 2024 Feb 29;12(3):190. doi: 10.3390/toxics12030190 (PMC10974976; doi:10.3390/toxics12030190)
Supplement: Supplementary file 1 [file toxics-12-00190-s001.zip › toxics-2876351-supplementary.pdf]

Table S1. Spearman's partial correlations controlling for age between plasma concentrations of HMGB1, sRAGE, ROS, ApoD and NRF2 and variables of AUD severity including age of alcohol use onset (OHONSET), age of alcohol dependence onset (OHDEPENONSET), duration of alcohol use (OHDURATION), duration of alcohol abstinence (OHABSTINEN) and periods of alcohol abstinence (OHNUMABS), among others.

Spearman's Partial Correlations

Variable		1	2	3	4	5	6	7	8	9	10	11	12	13	14	15
1. sRAGE	Spearman's rho	—														
	p-value	—														
2. ROS/RNS	Spearman's rho	0.129	—													
	p-value	0.252	—													
3. ApoD	Spearman's rho	0.150	-0.300	—												
	p-value	0.218	0.943	—												
4. NRF2	Spearman's rho	0.056	0.062	0.200	—											
	p-value	0.386	0.374	0.149	—											
5. HMGB1	Spearman's rho	0.117	-0.060	0.246	**0.381**	—										
	p-value	0.277	0.619	0.104	**0.023**	—										
6. OHONSET	Spearman's rho	-0.226	**-0.360**	0.109	0.085	0.057	—									
	p-value	0.861	**0.039**	0.301	0.342	0.396	—									
7. OHDEPEND	Spearman's rho	-0.231	-0.156	-0.055	0.252	-0.068	0.596	—								
	p-value	0.867	0.771	0.603	0.112	0.624	0.001	—								
8. OHABSTINEN	Spearman's rho	0.300	0.064	**0.411**	0.158	0.275	-0.056	-0.290	—							
	p-value	0.077	0.384	**0.023**	0.231	0.102	0.601	0.915	—							
9. OHNUMABS	Spearman's rho	**-0.340**	0.046	-0.154	0.018	-0.140	0.135	-0.127	0.130	—						
	p-value	**0.045**	0.411	0.773	0.466	0.748	0.269	0.718	0.278	—						
10. OHDURATI	Spearman's rho	**0.404**	0.109	-0.259	0.121	**0.398**	-0.251	-0.509	0.150	0.135	—					
	p-value	**0.018**	0.295	0.904	0.275	**0.022**	0.882	0.994	0.242	0.256	—					
11. COCONSET	Spearman's rho	-0.181	-0.214	0.050	-0.262	-0.347	0.468	-0.054	0.056	0.002	-0.040	—				
	p-value	0.713	0.748	0.439	0.794	0.866	0.102	0.555	0.443	0.497	0.547	—				
12. COCDEPEN	Spearman's rho	-0.109	-0.050	**0.503**	0.107	-0.149	0.457	0.189	0.387	-0.103	-0.454	0.693	—			
	p-value	0.632	0.561	**0.048**	0.370	0.677	0.108	0.313	0.152	0.618	0.920	0.006	—			
13. COCABSTINEN	Spearman's rho	0.206	-0.220	0.170	-0.095	0.275	-0.038	-0.225	-0.055	-0.375	0.157	0.008	-0.065	—		
	p-value	0.142	0.875	0.190	0.688	0.078	0.571	0.860	0.600	0.971	0.217	0.490	0.580	—		
14. COCNUMABS	Spearman's rho	0.153	-0.247	0.188	-0.164	0.010	-0.096	-0.009	-0.302	-0.399	-0.056	0.105	0.049	0.766	—	
	p-value	0.215	0.902	0.164	0.802	0.480	0.676	0.517	0.924	0.978	0.609	0.373	0.440	< .001	—	
15. COCDURATI	Spearman's rho	0.269	-0.149	0.209	-0.099	**0.339**	-0.192	-0.203	-0.062	-0.351	0.260	-0.483	-0.651	0.908	0.808	—
	p-value	0.079	0.780	0.138	0.696	**0.039**	0.822	0.834	0.612	0.961	0.095	0.944	0.989	< .001	< .001	—

Note 1: All tests one-tailed, for positive or negative correlations. *Note 2:* Conditioned on variables: Age.
